# The Relationship between Frontotemporal Effective Connectivity during Picture Naming, Behavior, and Preserved Cortical Tissue in Chronic Aphasia

**DOI:** 10.3389/fnhum.2016.00109

**Published:** 2016-03-16

**Authors:** Erin L. Meier, Kushal J. Kapse, Swathi Kiran

**Affiliations:** ^1^Department of Speech Language and Hearing Sciences, Aphasia Research Laboratory, Sargent College, Boston University, BostonMA, USA; ^2^Children’s National Medical Center, WashingtonDC, USA

**Keywords:** aphasia, oral picture naming, fMRI, effective connectivity, dynamic causal modeling, cortical damage, behavioral performance

## Abstract

While several studies of task-based effective connectivity of normal language processing exist, little is known about the functional reorganization of language networks in patients with stroke-induced chronic aphasia. During oral picture naming, activation in neurologically intact individuals is found in “classic” language regions involved with retrieval of lexical concepts [e.g., left middle temporal gyrus (LMTG)], word form encoding [e.g., left posterior superior temporal gyrus, (LpSTG)], and controlled retrieval of semantic and phonological information [e.g., left inferior frontal gyrus (LIFG)] as well as domain-general regions within the multiple demands network [e.g., left middle frontal gyrus (LMFG)]. After stroke, lesions to specific parts of the left hemisphere language network force reorganization of this system. While individuals with aphasia have been found to recruit similar regions for language tasks as healthy controls, the relationship between the dynamic functioning of the language network and individual differences in underlying neural structure and behavioral performance is still unknown. Therefore, in the present study, we used dynamic causal modeling (DCM) to investigate differences between individuals with aphasia and healthy controls in terms of task-induced regional interactions between three regions (i.e., LIFG, LMFG, and LMTG) vital for picture naming. The DCM model space was organized according to exogenous input to these regions and partitioned into separate families. At the model level, random effects family wise Bayesian Model Selection revealed that models with driving input to LIFG best fit the control data whereas models with driving input to LMFG best fit the patient data. At the parameter level, a significant between-group difference in the connection strength from LMTG to LIFG was seen. Within the patient group, several significant relationships between network connectivity parameters, spared cortical tissue, and behavior were observed. Overall, this study provides some preliminary findings regarding how neural networks for language reorganize for individuals with aphasia and how brain connectivity relates to underlying structural integrity and task performance.

## Introduction

Language is arguably one of the most advanced human cognitive functions, involving the ability to decode incoming messages and communicate complex thoughts in a variety of contexts. During the first 100 years of the study of language in the brain, topological theories of brain organization, which suggest that cognitive functions are mediated by specific, circumscribed neural regions, dominated the field (Mesulam, 1990; Catani and Mesulam, 2008; Duffau, 2008; Friston, 2011; Price, 2012; Duffau et al., 2014). These neural models of language originated from seminal work in patients with acquired left hemisphere brain damage by researchers in the 19th and 20th centuries such as Broca, Wernicke, and Geschwind. While the historical significance of such models should not be diminished, the actuality of neural organization is far less simplistic. Currently, neuroscientists have increasingly adopted and demonstrated support for a hodological view of neural organization in which specialized, anatomically segregated cortical regions demonstrate integrated functioning for successful task completion (Friston, 2011). In accordance with this view, the neuroimaging literature has shown that language processing involves a distributed neural network involving bilateral frontal, temporal and parietal regions (see reviews by e.g., Vigneau et al., 2006, 2011; Price, 2010, 2012).

Recent advances in neuroimaging techniques and data analysis methods have made investigations of the connectivity of language networks possible. Two such connectivity methods include functional connectivity, which captures the statistical relationships between activity in different neural regions, and effective connectivity, which measures the causal influence activity in specific regions exerts on other brain areas (Friston, 2009, 2011; Kahan and Foltynie, 2013). In general, connectivity analyses provide insight into time-dependent relationships between regions, and in the case of effective connectivity, these analyses allow researchers to determine the influence of regions of interest on each other and the influence of experimental tasks on activation in cortical hubs and the connections between these hubs. A burgeoning literature exists regarding task-based functional and effectivity connectivity for a variety of linguistic and speech processes in healthy adults, including speech perception (e.g., Griffiths et al., 2007; Chu et al., 2013), auditory processing (e.g., Saur et al., 2008, 2010), semantic and phonological processing (e.g., Bokde et al., 2001; Mechelli et al., 2003; Noppeney et al., 2006; Heim et al., 2009a,b; Seghier et al., 2011; Vandenberghe et al., 2013), repetition (e.g., Saur et al., 2008; Hartwigsen et al., 2013a), syntactic processing (e.g., Snijders et al., 2010; den Ouden et al., 2012), word generation and speech production (e.g., Vitali et al., 2005; Allen et al., 2008; Eickhoff et al., 2009; Papoutsi et al., 2009; Abel et al., 2011; Hartwigsen et al., 2013b; Smith et al., 2013; Rosso et al., 2014; Liljeström et al., 2015), and reading (e.g., Seghier and Price, 2010; Richardson et al., 2011). Such studies have provided insight into how different regions of the brain interact in the context of specific tasks and conditions in healthy individuals. For example, Noppeney et al. (2006) investigated category-specific differences in effective connectivity by manipulating presentation modality and task and discovered distinct patterns of bottom–up (i.e., category-specific responses to pictures in the ventral occipito-temporal cortex) and top–down effects (i.e., from prefrontal regions to left inferior-posterior middle temporal and anterior intraparietal regions for semantic decision tasks) during processing. Similarly, Allen et al. (2008) investigated context-dependent connectivity between left middle frontal and left middle temporal gyri during either suppression or initiation conditions of a sentence completion task and found that the connectivity strength between the two regions was increased for response suppression compared to response initiation.

While the number of task-based connectivity studies in normal language processing has risen dramatically in recent years (Friston, 2011), relatively little is known about the impact brain damage on left hemisphere regions has on functional integration of cortical regions for specific language tasks in persons with aphasia (PWA). A major area of debate within aphasia research pertains to the relationship between patterns of neural reorganization and successful behavioral recovery. Specifically, the role of regions that are involved in reorganization of function are frequently compared to the regions that may be involved in compensation of function (Kleim, 2011). This distinction is important when examining the role of hemispheric laterality for language and the degree of activation in specific left versus right hemisphere regions. While many language processes are left-lateralized, the right hemisphere is often active for certain language tasks (e.g., semantic processing) in neurologically intact individuals (Abel et al., 2011; Vigneau et al., 2011). In older neuroimaging studies, recruitment of right hemisphere homologues to left hemisphere language regions was linked to aphasia recovery (see reviews by Price and Crinion, 2005; Heiss and Thiel, 2006; Crosson et al., 2007; Thompson and den Ouden, 2008; Cappa, 2011), and individuals with large lesions especially have been shown to recruit more right hemisphere areas for language tasks (Turkeltaub et al., 2011). However, certain studies (e.g., Postman-Caucheteux et al., 2010) have found that over-activation of the right hemisphere for language tasks in PWA is maladaptive, and such findings suggest that the compensatory capabilities of the right hemisphere are limited (Heiss and Thiel, 2006; Crosson et al., 2007).

While the role of the right hemisphere with regards to language recovery or compensation is still in question, findings from recent research have emphasized the importance of undamaged left hemisphere regions in subserving language recovery. Specifically, successful recovery of language function after stroke is associated with increased ipsilesional activation in the left hemisphere. In a recent meta-analysis, Turkeltaub et al. (2011) concluded that recovery patterns and compensatory mechanisms in aphasia vary based on lesion location. Specifically, these authors found consistent activity for language tasks in preserved tissue in left hemisphere language regions, such as the left middle temporal gyrus (LMTG) and pars opercularis and triangularis in the left inferior frontal gyrus (LIFG). Moreover, additional activated nodes were seen in the aphasic data that had been recruited either to perform the role of a lesioned area (i.e., an additional node in LIFG triangularis), shifted slightly from an existing homologous healthy control node (i.e., LIFG pars orbitalis) or recruited to perform a different function [i.e., left middle frontal gyrus (LMFG)].

Based on this meta-analysis, it can be assumed that LIFG, LMTG, and LMFG are vital left hemisphere regions within the PWA language network, yet what is unknown is how these regions might interact for a specific language task such as picture naming. Based on normal psycholinguistic and neurocognitive models of word production, it is well understood that oral picture naming is a semantically driven process that includes stages related to retrieval of word meaning (i.e., lexical-semantic), retrieval of word form (i.e., lexical-phonological), and articulation (e.g., Dell et al., 1997; Levelt et al., 1999; Indefrey and Levelt, 2004; Schwartz et al., 2006). The neural correlates of the mechanisms underlying word production have been examined by a number of reviews by Indefrey and Levelt (2000, 2004; see also Indefrey, 2011); these authors posit that core processes of picture naming begin with conceptually driven lexical selection, mediated by activation in LMTG, which is then followed by subsequent phonological stages of processing. These authors note reliable activation in left posterior MTG (LpMTG) and left posterior superior temporal gyrus (LpSTG) for word form retrieval followed by activation in dorsal LIFG during phonological segmentation and syllabification. Overt picture naming also involves stages of phonetic encoding and articulation which have been mainly attributed to motor regions such as left precentral gyrus (LPCG), supplementary motor areas (SMA), and left anterior insula (Indefrey and Levelt, 2004).

The basic premise of the spatiotemporal patterns of word production described by Indefrey and Levelt (2000, 2004) is consistent with several other neuroimaging studies that have delineated stages of lexical-semantic processing and word retrieval; these studies also have confirmed the importance of LIFG and LMTG within the language network for neurologically intact individuals. Specifically, several studies (e.g., Binder et al., 2009; Binder and Desai, 2011; Turken and Dronkers, 2011; Visser et al., 2012) have suggested that LMTG is critical for heteromodal lexical-semantic processing. While LIFG has been found to play a central role in word-form (phonological) retrieval, it has also been implicated in semantic control processes such as correct selection of context-specific ambiguous word meanings, judgment of specific semantic features, and lexical selection when many competing representations are active (e.g., Thompson-Schill et al., 1997; Wagner et al., 2001; Devlin et al., 2003; Kan and Thompson-Schill, 2004; Badre et al., 2005; Saur et al., 2008; Spalek and Thompson-Schill, 2008; de Zubicaray and McMahon, 2009). In particular, Whitney et al. (2011) found that selective disruption of LIFG via transcranial magnetic stimulation resulted in poorer performance on executively demanding semantic tasks but not on non-semantic tasks, indicating that this region is important specifically for retrieval and control of semantic information within a large-scale language network. When considering the implications of damage to these regions in aphasia, it is important to highlight that word retrieval deficits in PWA have been attributed to either a deficit of access or control rather than a degeneration of underlying representations, particularly semantic representations (Jefferies and Lambon Ralph, 2006; Jefferies et al., 2007; Jefferies et al., 2008; Corbett et al., 2009a,b; Hoffman et al., 2011; Jefferies, 2013; Mirman and Britt, 2013; Thompson and Jefferies, 2013; Rogers et al., 2015). Consequently, the recruitment of core language regions such as LIFG and LMTG may differ based on the task at hand yet communication between these regions is likely critical for successful naming attempts in PWA in terms of *both* lexical-phonological retrieval and semantic processing, access, and control.

The third region that PWA consistently activated across studies in the Turkeltaub et al. (2011) meta-analysis was LMFG, a region that is not typically considered a “classic” language area like LIFG and LMTG. Like LIFG, though, regions in dorsolateral prefrontal cortex (including LMFG) have been implicated in executive control processes and are likely to be critical for picture naming, yet unlike LIFG, LMFG is associated with domain-general (i.e., non-language specific) cognitive control. LMFG is encompassed within the multiple demands network (also known as the task-positive or frontotemporal attention network) and is thought to mediate different types of behavior, including goal maintenance, selection of strategies for task completion, performance monitoring and other tasks (Fedorenko et al., 2013). In the context of language tasks, activation in LMFG has been associated with response selection or inhibition during semantically demanding tasks (Desmond et al., 1998; de Zubicaray et al., 2000; Collette et al., 2001; Jeon et al., 2009).

While several regions comprise the network involved in word retrieval and picture naming, the literature has shown that LIFG and LMFG play vital roles in lexical selection and control, and LMTG plays an important role in heteromodal semantic processing. However, how these regions interact with each other after stroke has not yet been examined. Understanding this interaction is particularly important as the role of left hemisphere engagement in recovery versus compensation is not well understood. For example, we do not know whether PWA network connectivity is driven by more intact, domain-general regions (such as LMFG) or by “classic” language regions (such as LIFG and LMTG) nor do we know if connectivity is driven by initial stages of lexical retrieval (e.g., semantic processing as mediated by LMTG) versus top–down control processes of selection (as mediated by LIFG or LMFG). At a broader level, it is also still unknown how brain damage and behavioral deficits are related to cortical interactions for a given task.

Therefore, the overall goal of this study was to examine frontotemporal effective connectivity induced by a picture naming task in PWA relative to healthy controls and to examine how connectivity parameters relate to behavioral performance and cortical damage in PWA. It should be noted that it was not the goal of this study to identify if or to what extent these regions are engaged in PWA relative to controls (which they presumably are). Rather, this study aimed to examine how a subset of critical regions within the PWA language network interact in order to better understand the mechanisms of language recovery after stroke. To examine this question, we employed dynamic causal modeling (DCM), a method which can be used to determine how coupling between regions and the direction of such effects are influenced by changes in the experimental tasks (Seghier et al., 2012, 2014). DCM is particularly advantageous to examine effective connectivity in stroke populations since modeling of region-specific hemodynamic response parameters can accommodate deviations from normal hemodynamic characteristics (Grefkes and Fink, 2011). DCM has been used to examine motor recovery in post-stroke patients (e.g., Grefkes et al., 2008, 2010; Rehme et al., 2011) as well as to examine changes in connectivity in aphasia as a function of rehabilitation (e.g., Abutalebi et al., 2009; Kiran et al., 2015). Additionally, DCM can be used to test specific hypotheses about the causal interactions between specific regions within a larger network. Consequently, as a preliminary investigation of PWA brain connectivity for picture naming, we studied the interactions of a simple, three-node network to better understand the integrated functioning of these core regions in PWA.

The specific goals of the study were as follows:

(1) The first aim was to investigate the nature of task-specific left hemisphere cortical reorganization in PWA relative to intact language networks in healthy individuals. More specifically, we were interested in investigating possible differences between PWA and controls in connectivity between posterior brain regions associated with basic-level semantic processing and prefrontal regions associated with semantic and general control processes for an overt picture naming fMRI task. We hypothesized that network activation in controls would be best explained by bottom–up models of processing (i.e., models in which LMTG acts as the driving region) as healthy individuals have intact semantic processing abilities and would need to rely less heavily on prefrontal regions to access lexical information. Conversely, we hypothesized that due to difficulty with accessing semantic and/or phonological information about target items, PWA data would align best with models of top–down processing (i.e., models with driving input to regions involved with lexical access and control mechanisms, LIFG and LMFG) as this task would induce more cognitive effort in PWA than in controls. Furthermore, we predicted that models with driving input to LIFG would best explain the data for PWA who had little damage to LIFG while models with driving input to LMFG would fit the data best for PWA with substantial damage to either LIFG or LMTG or to both regions.
(1) The second aim was to examine the relationships between connectivity parameters, cortical structural damage, and behavioral performance within the group of PWA. We predicted that greater strength of driving regions would be associated with better task performance and greater spared tissue in LIFG and LMTG in particular. Regarding connections, one possible hypothesis was that greater coupling from “classic” language regions (i.e., LIFG and LMTG) to other regions would be associated with greater spared tissue in those regions as well as better task accuracy. The alternative hypothesis was that greater spared tissue and better task performance would be associated with greater coupling from LMFG to other regions. This latter hypothesis was based on the premise that disconnection of these core hubs (as well as others) within the language network would result in a reliance on intact, domain-general regions like LMFG to modulate activation in intact tissue in perilesional areas.

## Materials and Methods

### Participants

The work reported here was part of a larger, multi-site project examining the neurobiology of language recovery in people with aphasia (NIH/NIDCD 1P50DC012283; PI: Cynthia Thompson)^1^ Twenty participants with chronic aphasia secondary to left hemisphere middle cerebral infarct (mean age = 62.26 years, 14 males) and 18 neurologically intact healthy controls (mean age = 59.09, 10 males) were recruited as part of this ongoing project. All behavioral testing was done according to and approved by the Boston University IRB and all imaging data were collected under and approved by the IRB at Massachusetts General Hospital. Neurological history and demographic information for all participants, including age, gender, handedness, and race and ethnicity, was collected via questionnaire. To be considered for the current study, participants had to meet the following criteria: no major neurological or psychiatric disorders (excluding stroke in the PWA group); primary language of English; adequate hearing; and normal or corrected-to-normal vision. Additional exclusionary criteria for PWA included onset of cerebral vascular accident (CVA) of less than 6 months from the time of study recruitment and multiple left-hemisphere CVAs. Within the larger sample, fMRI data were unusable for eight participants (two PWA, six controls) due to inability to complete the entire scan sequence (*n* = 1) or motion-related artifact. Ultimately, 13 PWA (mean age = 60.66 years, nine males) and 10 controls (mean age = 61.53, six males) showed activation for the fMRI task in the three regions of interest (see Effective Connectivity Analysis below) and were included in the final analyses (see **Tables 1A**,**B** for demographic information for PWA and controls, respectively).

**Table 1A:** Demographic information for PWA including age, gender, handedness, and months post onset of CVA (MPO).

	Patient participants			

ID	Age	Gender	Handedness	MPO
PWA1	56.28	M	R	17
PWA2	50.62	F	L	33
PWA3	78.39	M	R	13
PWA4	67.88	M	R	10
PWA5	55.32	M	R	138
PWA6	49.92	M	R	59
PWA7	72.01	F	R	39
PWA8	53.25	F	R	14
PWA9	42.75	M	R	19
PWA10	71.35	F	R	75
PWA11	50.00	M	R	71
PWA12	61.40	M	R	155
PWA13	79.39	M	R	12

Mean	60.66			50.38
Standard deviation	11.95			48.38
PWA14	81.91	M	R	12
PWA15	48.04	M	R	23
PWA16	63.92	F	R	65
PWA17	50.18	M	R	116
PWA18	78.83	M	R	25
PWA19	68.98	M	R	105
PWA20	64.72	F	R	26

Total Mean	62.26			51.35
Total standard deviation	12.16			45.32


*Portions of the table shaded in gray reflect information for participants excluded from the final analyses. Information for participants included in the final analyses is shown in white.*

**Table 1B:** Demographic information for control participants including age, gender, and handedness.

Control participants			

**ID**	**Age**	**Gender**	**Handedness**
C1	66.13	F	R
C2	66.83	M	R
C3	40.76	M	R
C4	54.76	F	R
C5	63.12	F	R
C6	68.97	F	R
C7	46.34	M	R
C8	75.94	M	R
C9	59.00	M	R
C10	73.49	M	R

Mean	61.53		
Standard deviation	11.41		
C11	24.13	M	R
C12	49.61	F	R
C13	62.48	M	R
C14	58.32	M	R
C15	76.76	F	R
C16	59.43	F	R
C17	48.25	M	R
C18	69.30	F	R

Total mean	59.09		
Total standard deviation	13.48		


*Portions of the table shaded in gray reflect information for participants excluded from the final analyses. Information for participants included in the final analyses is shown in white.*

Persons with aphasia were administered a battery of assessments to characterize the extent and severity of their language impairments. Standardized tests included the Western Aphasia Battery-Revised (WAB-R, Kertesz, 2007) to characterize the type and severity of aphasia as captured by the Aphasia Quotient (AQ); the Boston Naming Test (BNT, Kaplan et al., 2001) to determine confrontation naming abilities; and the three picture version of the Pyramids and Palm Trees Test (PAPT, Howard and Patterson, 1992) and the Word Semantic Association (subtest 51) of the Psycholinguistic Assessments of Language Processing in Aphasia (PALPA, Kay et al., 1992) to capture semantic association abilities. In addition, PWA were administered three picture-naming screening probes that consisted of 180 items across five categories (i.e., *birds*, *vegetables*, *fruit*, *clothing*, and *furniture*). See **Table 2** for the breakdown of test scores by participant.

**Table 2:** Performance on standardized language tests and other behavioral measures.

ID	WAB-R AQ	BNT (% acc)	PAPT (3 picture test % acc)	PALPA 51 (Total % acc)	Picture naming screener (avg % acc)
PWA1	87.20	81.67	96.15	76.67	47.22
PWA2	25.20	1.67	94.23	10.00	1.54
PWA3	74.10	86.67	94.23	60.00	65.12
PWA4	30.80	6.67	92.31	30.00	7.41
PWA5	48.00	10.00	88.46	40.00	14.81
PWA6	82.80	85.00	92.31	73.33	68.21
PWA7	95.20	75.00	96.15	86.67	46.60
PWA8	80.40	61.67	94.23	80.00	57.10
PWA9	92.70	71.67	94.23	70.00	46.60
PWA10	87.20	71.67	84.62	53.33	41.05
PWA11	33.60	1.67	78.85	10.00	0.93
PWA12	74.30	1.67	98.08	70.00	45.99
PWA13	26.90	ND	90.38	33.33	6.48

Mean	64.49	46.25	91.86	53.33	34.54
Standard deviation	27.18	37.65	5.28	26.21	24.72


*Performance on the baseline picture naming screener reflects averaged accuracy across three separate baselines, collapsed across categories. ND, no data.*

### fMRI Task and Stimuli

Persons with aphasia and controls completed two runs of an overt picture-naming task in the scanner. The experimental stimuli included color photographs of real items split across five categories (i.e., *birds*, *vegetables*, *fruit*, *clothing*, and *furniture*). All stimuli were concrete nouns balanced for familiarity, length, lexical frequency (CELEX, Van der Wouden, 1990) and concreteness^2^ (Coltheart, 1981). Across the two runs, each participant was administered 108 pictures from three of the aforementioned categories. All participants were administered items from the category *fruit*. For PWA, the other two categories were selected based on naming performance on the previously referenced picture naming screeners as these categories were then selected for treatment (which is outside the scope of this paper). For controls, the other two selected categories were counterbalanced across participants. Participants were required to name aloud each picture or say “skip” for the pictures they were unable to name as soon as they saw the stimulus. Control stimuli were 36 pixelated, scrambled pictures of the experimental stimuli, split equally across both runs. Participants were required to say “skip” for each of the control items as soon as they saw the stimulus.

**FIGURE 1:**
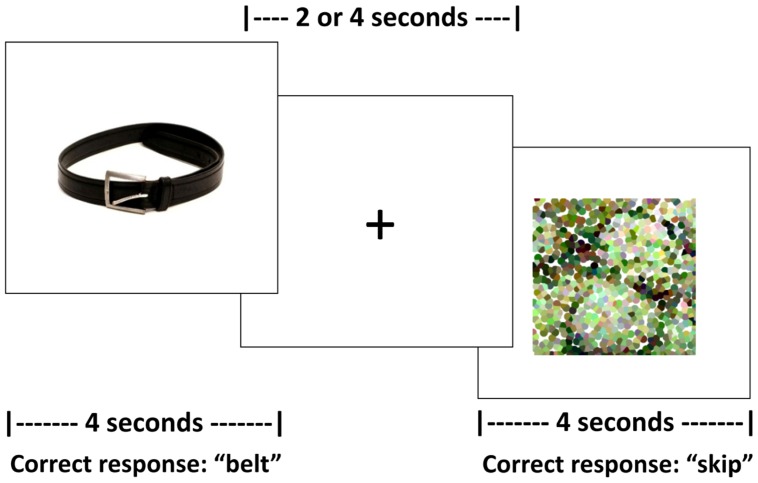
**Schematic of the fMRI picture naming task**.

For this task, an event-related design with jittered, randomized inter-stimulus intervals (ISIs) between 2 and 4 s was employed. Jittered ISIs can compensate for brief motion artifacts related to speaking, increase statistical efficiency of the experimental design, and have been implemented in previous studies that do not use sparse sampling but require overt responses (Birn et al., 2004). During the ISI, a fixation of a “+” appeared on the screen; participants were instructed to respond before, not during, the ISI. Trial duration was 4 s for both experimental and control stimuli. Task duration for each run was 8 min and 24 s. See **Figure 1** for a schematic of the task.

### fMRI Data Acquisition

Magnetic resonance images were acquired at the Athinoula A. Martinos Center for Biomedical Imaging in Charlestown, MA on a 3T Siemens Trio Tim using a 20-channel head + neck coil. T1 structural images were collected using the following parameters: 176 sagittal slices, 1 mm × 1 mm × 1 mm voxels, 240 × 240 matrix, FOV = 240 mm, flip angle = 9, fold-over direction = AP, TR = 2300 ms, TE = 2.91ms. For each run of the picture naming task, blood-oxygen-level-dependent (BOLD) functional images were acquired with the following parameters: interleaved, parallel acquisition; 40 axial slices, 3 mm thick; 2 × 2 × 3 mm voxels; 0.3 mm interslice gap; 80 × 78 matrix; FOV = 240 mm; flip angle = 90, fold-over direction = AP, TR = 2570 ms, TE = 30 ms. Verbal responses were recorded with a Fibersound Fiber Optic microphone (Micro Optics Technologies, Cross Plains, Middleton, WI, USA).

### fMRI Data Analysis

#### Preprocessing

**FIGURE 2:**
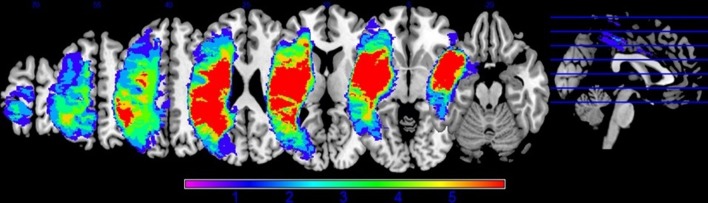
**Lesion overlap of all thirteen PWA included in the DCM analysis**.

**FIGURE 3:**
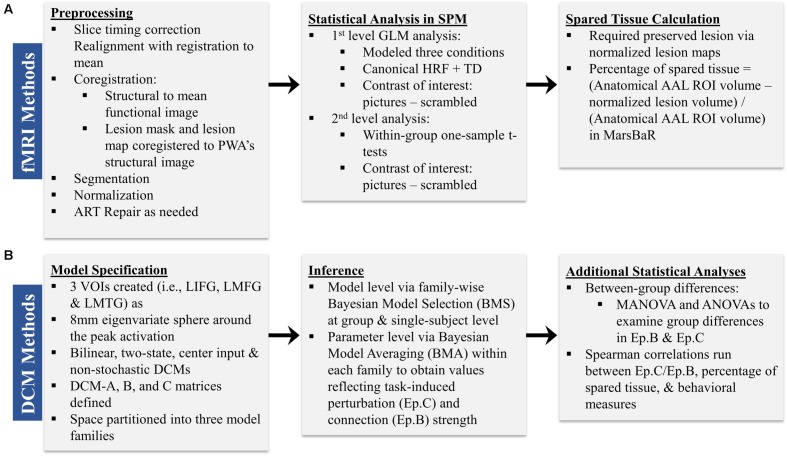
**Overview of the sequence of (A) fMRI and (B) DCM methods**.

Preprocessing was performed to account for participant motion and physiological fluctuations, remove slow baseline drifts, and correct for timing of image acquisition. Data were analyzed using SPM8 software in the following sequence (Wellcome Trust Centre for Neuroimaging, 2009). First, slice timing correction was applied with reference to the middle slice. Motion correction using the Realign function was employed. After realignment, structural T1 images were coregistered to the mean functional image. For PWA, lesion masks were manually drawn in MRIcron^3^ using each participant’s T1 image (see **Figure 2** for lesion overlap across the group of PWA); these lesion masks (i.e., in which the lesion was deleted) as well as lesion maps (i.e., in which the lesion was preserved) were also coregistered to the T1 structural image. Next, unified segmentation of the coregistered structural images into white matter, gray matter, and cerebral spinal fluid was performed. For the PWA data, the coregistered lesion mask was included so that lesioned regions (which contained a value of zero) would be excluded during the estimation of segmentation parameters (Brett et al., 2001; Meinzer et al., 2013). Structural and functional images were spatially normalized to the Montreal Neurological Institute (MNI) template in SPM8. Correction for slow baseline drifts was done using a high-pass filter with a cutoff of 1/128 s. Spatial smoothing of the functional data was not performed as smoothing can result in compromised accuracy of activation localization, which can be particularly problematic in individuals with structural damage (Meinzer et al., 2013). In addition to Realign, the ArtRepair toolbox in SPM8 was employed as needed to account for potential movement-related artifacts in the data by repairing via linear interpolation large variations (i.e., >0.5 mm) in volume-to-volume motion (Mazaika et al., 2009). See the first panel in **Figure 3A** for an overview of the preprocessing pipeline employed.

#### Statistical Analysis in SPM

##### First-level analysis

First-level analysis was performed based on the General Linear Model (GLM) in SPM8. Stimulus onsets and durations were convolved with the canonical hemodynamic response function (HRF) and its temporal derivative. Conditions included pictures, scrambled pictures, and fixation. Each condition was modeled separately for each run and then runs were concatenated within the GLM. Motion correction parameters obtained during realignment were included in the model as regressors. Model parameters were estimated using a restricted maximum likelihood (ReML) approach, and serial correlations were specified using an AR (1) error model. The main contrast of interest across participants was *pictures (experimental)* – *scrambled pictures (control)*. Activation maps reflected activation across concatenated runs and were thresholded at the family wise error rate (F.W.E.). If activation was not seen at the F.W.E. threshold, uncorrected activation maps (*p* < 0.001) were obtained. Ultimately, uncorrected activation was used in the connectivity analyses for all individuals in order to stay consistent across participants. Anatomical labels for active regions were obtained by entering the coordinates of active voxels into the Anatomy Toolbox, v.17, in SPM8. See the second panel in **Figure 3A** for an overview of the main components of the first level analysis.

##### Second-level analysis

Second-level analyses were performed to identify within-group whole brain activation for the contrast of interest. Specifically, one-sample *t*-tests showing activation for *pictures (experimental)* > *scrambled pictures (control)* were obtained for each group at an uncorrected threshold of *p* < 0.005. It should be noted that these analyses were performed to reveal activation at the group level yet the activations from the single-subject GLMs were used in the creation of the volume of interest (VOI) spheres used in the DCM analyses (see Model Specification below).

#### Calculation of Spared Tissue

In accordance with the second aim of the present study, the amount of spared tissue within the three regions included in the DCMs was calculated for each PWA in order to investigate the relationship between the underlying structural integrity of these regions and the direction and strength of the inter-regional connections. For each PWA, anatomical regions of interest (ROIs) corresponding to LIFG, LMFG, and LMTG were created using the AAL atlas within the MarsBaR toolbox in SPM8 (Brett et al., 2002). A lesion map, in which the lesioned voxels were given a binary 1 rather than 0, was normalized from native to MNI space, and this map was subtracted from each of the ROIs generated in MarsBaR to yield the volume of spared tissue per ROI. The percentage of spared tissue in each region was calculated by dividing the volume of spared tissue by the total volume of the AAL atlas ROI (see the third panel of **Figure 3A**). This allowed for a uniform comparison of lesioned tissue within ROIs across participants.

### Effective Connectivity Analysis

Effective connectivity was analyzed with the DCM10 toolbox in SPM8. DCM is a hypothesis-driven method that employs differential equations to model and infer directionality of context-dependent inter-regional interactions (Friston et al., 2003; Penny et al., 2004; Seghier et al., 2010; Stephan et al., 2010). External inputs, such as a task of interest, cause change at a neuronal level which in turn causes changes in the BOLD signal. In DCM, the observed changes in the hemodynamic response are then linked to hidden neuronal states via an empirical forward model, which allows for inferences about direct neural activity. Based on an *a priori* hypothesis, researchers define a model space which specifies regions of interest and the connections between those regions. The neurodynamics of the system are modeled by bilinear state equations, in which three parameters are estimated: the intrinsic connections or interactions between regions within a given model in the absence of input (DCM-A matrix); the modulatory effect on the connections between regions secondary to external inputs (DCM-B matrix); and the direct effect of external inputs to a given region (DCM-C matrix). According to their goals, researchers can make inferences on the structure of given model(s) or on the estimated model parameters themselves (i.e., Ep.A, Ep.B, Ep.C values, corresponding to the aforementioned parameters of the state equation) (Stephan et al., 2010).

#### Model Specification

In the present study, we constructed a model space (i.e., the total number of models defined per participant) including the three left hemisphere regions Turkeltaub et al. (2011) identified as vital to language processing in PWA that mediate various aspects of picture naming: LIFG, LMFG, and LMTG. The neuroimaging literature provides a solid basis for the utilization of a hypothesis-driven method for investigating connectivity for oral picture naming, as noted in the Introduction. Furthermore, tractography studies in humans have shown that extensive white matter connections exist between dorsolateral prefrontal cortex, ventrolateral prefrontal cortex, and mid-temporal regions. Specifically, portions of the superior longitudinal fasciculus (SLF) (including the lateral portion and the arcuate fasciculus) connect posterior-inferior temporal regions to the frontal operculum and constitute a dorsal pathway that is linked to phonological processing (Catani et al., 2002, 2005; Frey et al., 2008). Pathways associated with semantic processing include the inferior occipito-frontal fasciculus (IFOF), which connects posterior-inferior temporal cortex to the dorsolateral prefrontal cortex and orbito-frontal regions; the middle and inferior longitudinal fasciculi, which run laterally along the temporal cortex and connect intratemporal regions (e.g., anterior temporal lobe (ATL) with MTG); and the uncinate fasciculus (UF), which links ventral prefrontal cortex (i.e., orbito-frontal regions) with the ATL (Catani et al., 2002, 2005; Parker et al., 2005; Catani and Mesulam, 2008; Glasser and Rilling, 2008; Saur et al., 2008; Turken and Dronkers, 2011; Binney et al., 2012; Cloutman and Lambon Ralph, 2012; Sarubbo et al., 2013). Additionally, association U-fibers connect pars opercularis, pars triangularis, and pars orbitalis in LIFG and connect the frontal operculum to the dorsolateral prefrontal cortex (Binney et al., 2012; Lemaire et al., 2013). It should be noted that lack of anatomical connectivity does not preclude functional connections for language (see Friston, 2011; Cloutman and Lambon Ralph, 2012), yet the presence of such robust structural connections provides a basis for understanding how functional connections may still exist in the presence of damage to left hemisphere brain regions.

**FIGURE 4:**
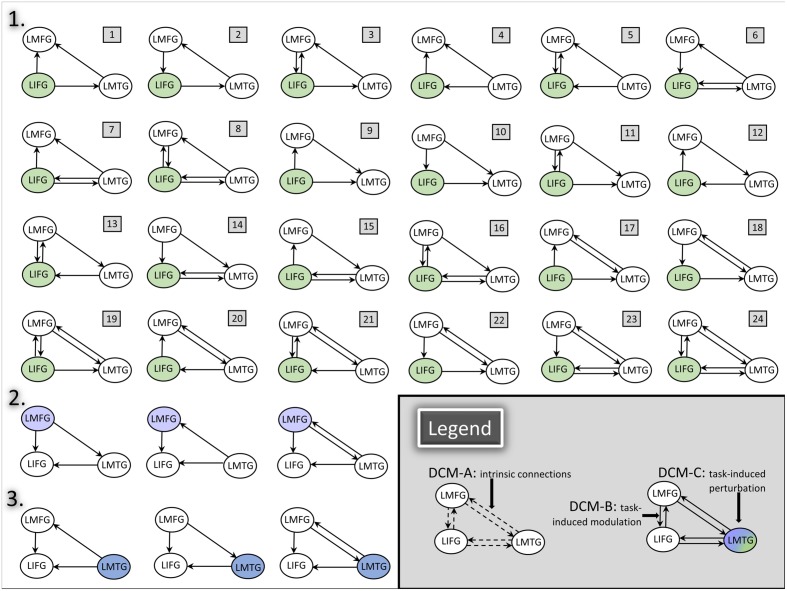
**DCM model space.** Full, bidirectional endogenous connections between all regions were modeled in DCM-A. For each model, driving input to only one region was modeled in DCM-C. All possible combinations of uni- and bidirectional modulations were modeled across the model space; for each model, the input region modulated at least one other region in DCM-B. The full model space for all 24 models in Family #1 is schematized in the figure above **(1)**. Family #2 included models with the same modulatory connections as Family #1 with three additional models **(2)** and excluding models #1, #4, and #7 due to lack of modulation from LMFG to the other two regions. Similarly, Family #3 included models with the same modulatory connections as Family 1 with three additional models **(3)** and excluding models #9, #10, and #11 due to lack of modulation from LMTG to the other two regions.

Consequently, full, bidirectional intrinsic connections were specified in the DCM-A matrix. Relatively little is known regarding the exact mechanisms of task-based dynamic reorganization for picture naming in PWA. Therefore, in order to study the direct effect of the picture naming task on specific regions and the subsequent modulation between regions, models with driving input to one of the three regions (i.e., LIFG, LMFG, LMTG) with all possible combinations of uni- and bidirectional modulation between regions were specified in the DCM-C and DCM-B matrices. Models were excluded from the model space if driving input was to a given region (e.g., LIFG) but that region was not modeled as modulating activity in either of the other regions. Consequently, the final model space contained a total of 72 models. We hypothesized that the differences in exogenous input to regions would alter network activity, and to test that hypothesis, the model space was further partitioned into three families which differed based on the input to region (i.e., LIFG, LMFG, or LMTG). The partitioning resulted in three families with 24 models per family (see **Figure 4** for a schematic of the model space).

For each participant, VOIs from each of the three regions were extracted from each run as 8 mm eigenvariate spheres around the most significantly active voxel within the region and adjusted for F-contrast effects of interest. Of note, spatial localization of VOIs was restricted to the anatomical boundaries of the region as defined by the AAL atlas. One VOI for LIFG was created around the peak maxima in either pars triangularis, pars orbitalis or pars opercularis. No other criteria regarding VOI selection and creation were applied. The reason for this methodology was twofold: first, in order to do group-level comparisons, the same models with the same regions had to be specified for each individual within each group. Due to the variable sizes and locations of lesions within given anatomical regions for PWA, peak maxima were expected to differ from PWA to PWA. Consequently, using more stringent VOI selection criteria would have led to the exclusion of certain PWA from the analyses. Second, similar loose VOI selection criteria were applied for the control group in order to stay consistent across participant groups. After VOI creation, DCMs according to the aforementioned model space were constructed for each participant for individual and group-level analyses. The basic components of model specification are shown in the first panel of **Figure 3B.**

#### Inferences at the Model and Parameter Level

Following model specification and estimation, we analyzed the connectivity data in separate stages (see the second panel of **Figure 3B**). First, we applied a random effects family wise Bayesian Model Selection (BMS) to understand the pathophysiological mechanism versus normal mechanism utilized to perform the task in the PWA and control groups, respectively, and to ascertain which family of models best fit each set of data at both the single-subject and group levels (Stephan et al., 2009; Penny et al., 2010; Stephan et al., 2010). In random effects analysis, the inference is made on the posterior estimates of the model frequency, and the exceedance probability (i.e., xp value) reflects the belief that one model (or set of models for family level comparisons) is more likely than any other in the model space to fit the data (Stephan et al., 2009). Second, we employed Bayesian Model Averaging (BMA) within each set of families; this type of BMA analysis utilizes the distribution of priors across all models within a family to determine each set of family specific coupling parameters (Penny et al., 2010). Next, we analyzed the model parameters by entering subject-wise Ep.B and Ep.C values into MANOVA/ANOVAs in order to determine the differences within and between groups on the driving and modulatory influences of task-induced activity on regions and connections within the model space. Lastly, as described below, we used single-subject Ep.B and Ep.C values to investigate the relationship between brain connectivity and other metrics in the patient group (see the third panel of **Figure 3B**).

### Relationship between Connectivity, Lesion Characteristics, and Behavior

Specifically, we investigated the relationship between connectivity parameters, the amount of spared cortical tissue in each of the three regions, and behavioral performance on naming tasks in the PWA group. The same six connections (i.e., LIFG→LMFG, LIFG→LMTG, LMFG→LIFG, LMFG→LMTG, LMTG→LIFG, and LMTG→LMFG) were modeled in each family, and it was our working hypothesis that the coupling strengths of these connections differed according to the exogenous input driving network modulation. Therefore, we first conducted another MANOVA within the PWA group to determine if coupling parameters (i.e., Ep.B values) differed significantly between families. Due to the non-normal distribution of the data, Spearman correlations were used for all analyses. In the first set of analyses, correlations were obtained between percentage of spared cortical tissue and Ep.B values (i.e., the strength of modulatory connections between regions) as well as between spared tissue and Ep.C values (i.e., the strength of the driving input to a region). In the second set of analyses, we correlated Ep.B and Ep.C values with behavioral performance on the fMRI task and the picture naming screeners in order to determine the relationship between network connectivity and naming abilities.

## Results

### Percentage of Spared Cortical Tissue and Behavioral Results

Results of the calculations of percent spared cortical tissue in LIFG, LMFG, and LMTG are shown in **Table 3.** As a group, PWA had the most spared tissue in LMFG, and only one participant (i.e., PWA 10) had less spared tissue in LMFG compared to the amount of spared tissue in the other two regions. As a group, the least spared tissue was seen in LMTG yet the relative preservation of LMTG and LIFG differed from participant to participant with some participants presenting with more anterior lesions (e.g., PWA 13), other participants presenting with more posterior lesions (e.g., PWA 3) and others presenting with damage to both anterior and posterior regions (e.g., PWA 2, PWA 12).

**Table 3:** Percentage of spared tissue in LIFG, LMFG, and LMTG across PWA.

	LIFG	LMFG	LMTG
PWA 1	96.60	100.00	79.36
PWA 2	65.51	96.26	68.09
PWA 3	99.05	100.00	33.51
PWA 4	80.25	100.00	14.16
PWA 5	92.47	96.44	70.38
PWA 6	89.59	100.00	78.15
PWA 7	99.98	100.00	93.91
PWA 8	100.00	100.00	91.80
PWA 9	99.98	100.00	97.09
PWA 10	80.77	73.95	99.66
PWA 11	49.15	51.04	12.55
PWA 12	58.68	98.66	46.11
PWA 13	53.89	98.75	98.92

TOTAL AVG	81.99	93.47	68.05


*Heat map colors reflect the amount of preserved tissue in each region such that green greatest preservation to red greatest damage.*

Analysis of overt naming responses from the fMRI task revealed that naming accuracy was significantly lower for PWA than for controls [PWA mean: 26.05%, control mean: 60.30%; *t*(17) = -3.87, *p* < 0.001). In the PWA group, greater spared tissue in LIFG was significantly related to better accuracy on the fMRI task (*r* = 0.682, *p* < 0.05) but no significant relationships between spared tissue in LMFG or LMTG and the fMRI task were found. However, greater spared tissue in both LIFG and LMFG was significantly associated with better accuracy on the averaged naming screeners (*r* = 0.741, *p* < 0.01 and *r* = 0.748, *p* < 0.01, respectively). As there was overlap in the items included in the fMRI task and the naming screener (i.e., the fMRI task included a subset of items from three of the five categories of the full naming screener), naming screener accuracy served as a proxy for naming abilities under normal circumstances without the constraints of the fMRI task conditions.

### Whole Brain Activation

For analysis of the fMRI data, all task trials were included in the first-level GLM. Each of the 13 PWA and 10 controls whose data were used in the DCM analyses showed activation in each of the three regions of interest at the uncorrected threshold (*p* <0.001) for the contrast *pictures (experimental)* – *scrambled pictures (control)*. The MNI coordinates of the most active voxels in each region of interest for each participant that were used in creation of VOIs for the DCMs are shown in **Table 4.**

**Table 4:** MNI coordinates of the suprathreshold active voxels in each region of interest for the *pictures – scrambled pictures* contrast used in the DCM analysis.

	LIFG				LMFG				LMTG			
			
ID	*x*	*y*	*z*	Cluster size	*x*	*y*	*z*	Cluster size	*x*	*y*	*Z*	Cluster size
PWA1	-60	12	9	2	-39	9	51	1	-60	3	-15	1
PWA2	-42	21	-3	1	-36	57	21	1	-51	-66	0	1
PWA3	-57	24	3	1	-39	15	54	3	-60	-39	0	1
PWA4	-45	15	18	5	-42	33	33	1	-63	-45	9	1
PWA5	-45	30	21	5	-24	54	12	2	-57	-24	-3	1
PWA6	-36	39	15	5	-45	15	39	4	-45	-60	-3	9
PWA7	-54	36	12	2	-27	42	15	1	-51	-21	-18	1
PWA8	-39	21	9	3	-27	6	48	1	-66	-9	-12	1
PWA9	-48	36	9	1	-48	12	42	10	-51	-69	3	5
PWA10	-48	30	21	3	-24	27	54	1	-60	-39	6	1
PWA11	-36	30	21	1	-30	63	3	1	-66	-9	-3	1
PWA12	-51	33	15	14	-30	6	60	2	-60	-15	-24	1
PWA13	-54	9	18	1	-33	51	9	1	-60	0	-18	2
C1	-51	33	12	795	-27	51	9	795^∗^	-63	-36	3	8
C2	-48	24	30	14	-24	6	57	4	-51	-60	6	4
C3	-54	18	33	10	-48	15	48	2	-60	-36	3	1
C4	-36	27	18	1	-45	6	54	2	-51	-27	0	1
C5	-39	33	12	22	-21	21	48	1	-57	-63	-3	19^∗^
C6	-48	21	-9	7^∗^	-30	0	57	2	-45	-75	18	86^∗^
C7	-48	21	30	141	-36	48	33	1	-66	-30	0	22
C8	-36	15	27	15	-27	6	57	1	-60	-51	-9	1
C9	-48	48	12	15	-33	21	30	36	-54	-6	-15	6
C10	-45	30	21	18	-30	0	57	1	-54	-66	0	1


*The ^∗^ reflects the size of a cluster for which the peak maxima of the cluster is in a different anatomical region. VOIs were created with the peak maxima that fell within the anatomical boundaries of the regions of interest.*

**FIGURE 5:**
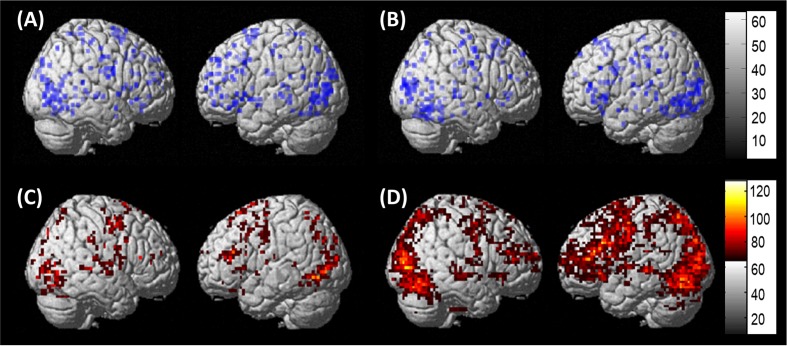
**Whole brain activation. (A)** Results of the one-sample *t*-test in PWA at uncorrected (*t* = 3.05, *p* < 0.005) for *pictures > scrambled pictures*. **(B)** Results of the one-sample *t*-test in controls at uncorrected (*t* = 3.25, *p* < 0.005) for *pictures > scrambled pictures*. **(C)** Overlap of the 13 individual PWA activation maps at uncorrected (*p* < 0.001), cluster size of 3 voxels for *pictures* – *scrambled pictures*. **(D)** Overlap of the 10 individual control activation maps at uncorrected (*p* < 0.001), cluster size of 5 voxels *pictures* – *scrambled pictures.*

Results of the second-level, one-sample t-tests revealed that each group showed activation in each of the regions of interest for *pictures (experimental)* > *scrambled pictures (control)* at an uncorrected threshold (PWA: *t* = 3.05, *p* < 0.005; controls: *t* = 3.25, *p* < 0.005). Of note, similar group-level activation was seen in bilateral frontal, temporal and parietal regions in each group (see **Figures 5A,B**). As single-subject activation from the first-level analysis was used in DCM, overlays of individual activation maps for each group also were visualized using the xjView toolbox in SPM8^4^ and are shown in **Figures 5C,D.**

### Family Wise BMS

**FIGURE 6:**
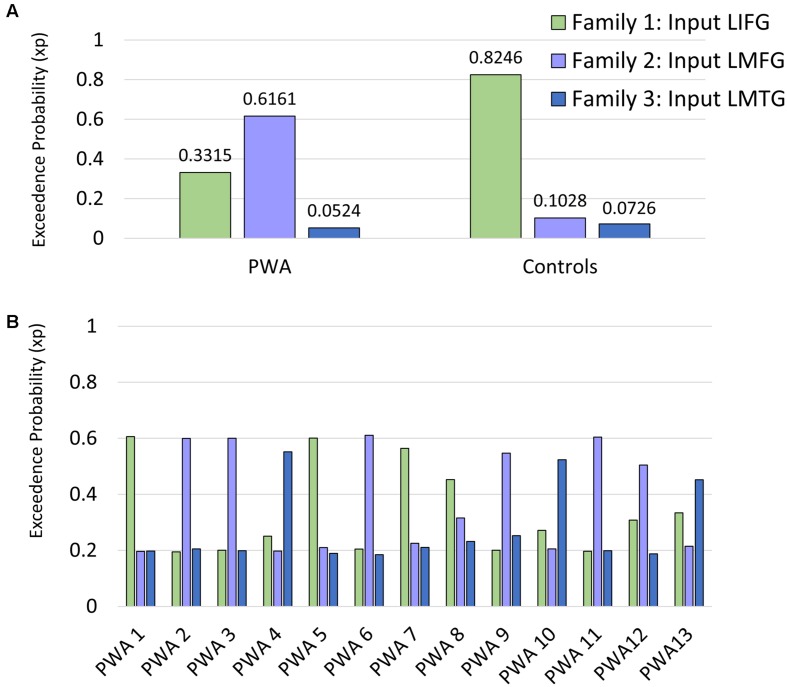
**Family wise BMS. (A)** Group-level family wise BMS results. **(B)** Single-subject family wise BMS for the PWA.

Per the first aim of the study, differences between groups in network connectivity were investigated. Comparison of the three model families within each group revealed that the family of models that best fit the data differed between groups. Specifically, model family #1, which included driving input to LIFG, was the winning family for control participants (xp = 0.825) while model family #2, which included driving input to LMFG, was the winning family for PWA (xp = 0.616) (see **Figure 6A**). As xp values are probability values, a value of 1.0 would indicate 100% probability that the family of models exceeds the expected probability of explaining the data. Given the low xp value for family #2 for the group of PWA, individual family wise BMS results were examined to see why family #2 was not the overwhelming winner across the group. Indeed, these results confirm the heterogeneity of the PWA sample as only six of the 13 PWA demonstrated the group-level pattern of best model family fit for family #2 (see **Figure 6B**). Next, we further investigated the differences in connectivity parameters between both participant groups as well as within the group of PWA.

### Connectivity Parameters from BMA

In general, positive coupling strength indicates that the modulatory region promoted activation in the other region while negative coupling strength indicates that the modulatory region exerted an inhibitory influence on activation in the other region. Similarly, larger and more positive values for strength of driving input to regions indicate that the task of interest exerted greater perturbation of activation in that region. The greater the absolute value of the parameter, the greater the effect of the task on that connection/region; consequently, a value of 0 Hz is indicative of no effect of the task on the connection/region. In consideration of the family wise BMS results, we examined differences in BMA parameters between groups by family in terms of strength of modulatory connections (i.e., Ep.B) via a one-way MANOVA with group (i.e., PWA/control) and family (i.e., input to LIFG, LMFG, LMTG) as the independent variables and Ep.B values for each connection as the dependent variables. We examined differences in input to driving regions (i.e., Ep.C) via a 2 (group) × 3 (family/input region) ANOVA.

The overall model explaining group differences in task-induced modulatory effects on connections was not significant [*Pillai’s trace* = 0.107, *F*(6,58) = 1.16, *p* = 0.341], and while the main effect of family was significant [*Pillai’s trace* = 0.857, *F*(12,118) = 7.37, *p* < 0.001], the group × family interaction did not reach significance [*Pillai’s trace* = 0.243, *F*(12,118) = 1.36, *p* = 0.195]. However, if coupling strength differed between groups for only certain connections, it is likely that including all data within one model diluted these differences. Therefore, we next examined between-group differences in Ep.B values for each connection via 2 (group) × 3 (family) ANOVAs. The only statistically significant difference between controls and PWA was found in the strength of the modulatory effect of LMTG on LIFG [*F*(1,63) = 6.75, *p* < 0.05], and while the main of effect of family approached significance (*p* = 0.055), a significant interaction of group by family was not found (*p* = 0.547). These results indicate that coupling strength of this connection differed between controls and PWA across families. Specifically, coupling from LMTG to LIFG was significantly more negative for controls relative to a nearly null effect of task on the connection for PWA (-0.031 Hz for controls, -0.009 Hz for PWA). With regards to Ep.C values (i.e., strength of exogenous input to driving regions), no significant between-group differences were seen [*F*(5,63) = 1.44, *p* = 0.713] nor was the group by family (i.e., input region) interaction significant (*p* = 0.533). These results indicate that overall strength of driving input did not differ significantly between groups by driving region. These collective results indicate that PWA and controls did not differ with regards to the strength of driving input to regions. They did differ in terms of the connection from LMTG to LIFG such that LMTG exerted an inhibitory influence on activation in LIFG for controls whereas minimal task-induced modulation of LMTG on LIFG was observed for PWA.

### Relationship between Connectivity Parameters, Spared Cortical Tissue, and Behavior in PWA

In line with the second aim of the study, we next investigated the relationship between connectivity, structural integrity, and behavior within the PWA group. First, we conducted a one-way MANOVA that included family as the independent variable and connection strength values (i.e., Ep.B) as the dependent variables to determine if strength of connections differed significantly between families. Once again, the rationale for this analysis was that we hypothesized that coupling parameters between the same connections would differ between families as exogenous input to regions would modify network connectivity. Indeed, this analysis showed that the overall main effect of family was significant for PWA [*Pillai’s trace* = 1.10, *F*(12,64) = 6.56, *p* < 0.001], and the strength of each connection excluding LMFG→LMTG [*F*(2,36) = 2.37, *p* > 0.05] differed significantly from family to family [LIFG→LMFG: *F*(2,36) = 6.11, *p* < 0.01; LIFG→LMTG: *F*(2,36) = 5.71, *p* < 0.01; LMFG→LIFG: *F*(2,36) = 5.15, *p* < 0.05; LMTG→LIFG: *F*(2,36) = 4.59, *p* < 0.05; LMTG→LMFG: *F*(2,36) = 4.08, *p* < 0.05]. Due to these results, all subsequent analyses involving Ep.B values were conducted for each family separately. In the next set of analyses, using Spearman correlations, we examined the relationships between (1) connectivity parameters (i.e., Ep.B and Ep.C values) and the percentage of spared cortical tissue in each region of interest (i.e., LIFG, LMFG, and LMTG) and (2) connectivity parameters and behavioral performance on language measures (i.e., accuracy on the fMRI task and the averaged naming screener performance).

#### Relationship between Connectivity Parameters and Spared Cortical Tissue

First, we examined the relationship between task-induced connection (Ep.B) and driving input strength (Ep.C) per family and the amount of spared cortical tissue in LIFG, LMFG, and LMTG. For family #1 (i.e., models with input to LIFG), a significant moderate negative correlation between the amount of spared tissue in LIFG and the connection from LMFG to LIFG was found (*r* = -0.580, *p* < 0.05) such that the greater the spared tissue in LIFG, the more negative the task-induced coupling was from LMFG to LIFG. Similarly, a significant correlation was found between spared tissue in LMFG and the strength of the connection from LMFG to LIFG (*r* = -0.627, *p* < 0.05) such that the more LMFG was preserved, the more negative the coupling was from LMFG to LIFG. A different pattern was observed for the only significant correlation for family #2 (i.e., models with input to LMFG) such that the more spared tissue in LMTG, the more positive the coupling was from LMTG to LIFG (*r* = 0.731, *p* < 0.01). In fact, nearly preserved LMTG was associated with Ep.B values approaching 0 Hz, indicative of a null effect of task on the connection. For family #3 (i.e., models with input to LMTG), negative correlations similar to the family #1 results were found. Specifically, the greater the spared tissue in LMFG, the more negative the coupling was from LMFG to LMTG (*r* = -0.729, *p* < 0.01) and the more spared tissue in LMTG, the more negative the coupling was from LMTG to LMFG (*r* = -0.643, *p* < 0.05).

**FIGURE 7:**
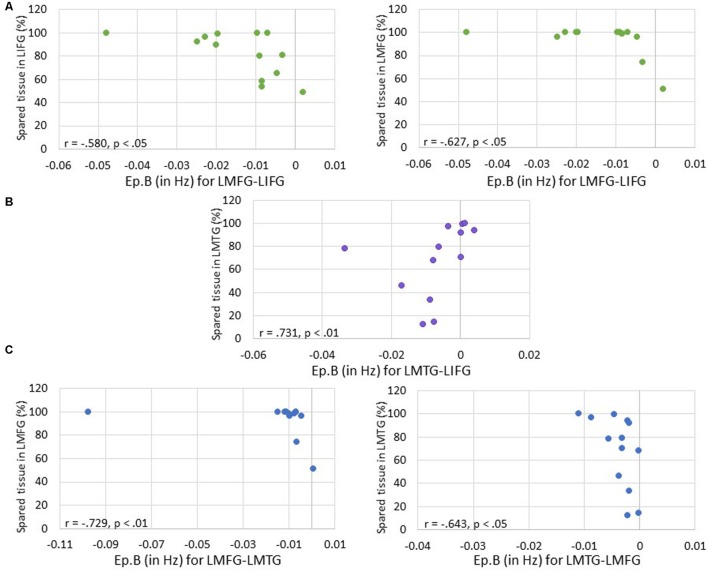
**Correlations between percentage of spared tissue and strength of the connections (i.e., Ep.B values in Hz). (A)** For family #1, significant correlations were found between the connection strength of LMFG → LIFG and the percentage of spared tissue in LIFG (shown on the left) and LMFG (shown on the right). **(B)** For family #2, a significant correlation was found between the connection strength of LMTG → LIFG and the amount of spared tissue in LMTG. **(C)** For family #3, significant correlations were found between the connection strength of LMFG → LMTG and percentage spared tissue in LMFG as well as the connection LMTG → LMFG and the amount of spared tissue in LMTG.

**FIGURE 8:**
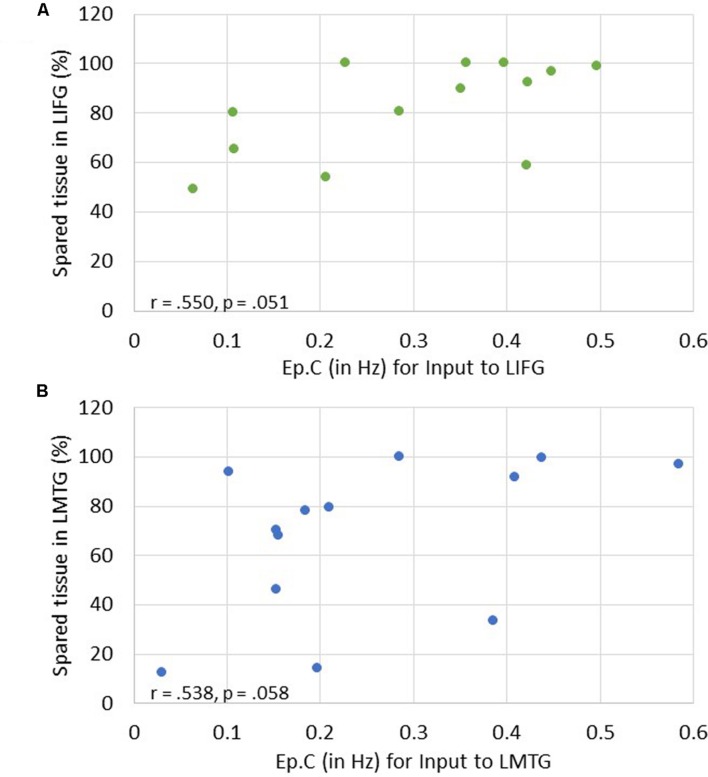
**Correlations between percentage of spared tissue and strength of task-induced perturbation to specific regions (i.e., Ep.C values in Hz). (A)** For family #1, an association that approached significance was found between strength of driving input of LIFG and amount of spared tissue in LIFG. **(B)** For family #3, a trending association between driving input strength of LMTG and the amount of spared tissue in LMTG.

Of note, it is possible that the outlier seen in **Figures 7A,C** may have primarily driven these results. As such, when this outlier was removed, the relationship between percentage of spared tissue in LIFG and the connection from LMFG to LIFG for family #1 was no longer significant (*r* = -0.531, *p* = 0.075), and the relationship between the amount of spared tissue in LMFG and the connection from LMFG to LMTG for family #3 only approached significance (*r* = -0.559, *p* = 0.059). All other correlations remained significant. Also, as is apparent in **Figure 7**, many of the Ep.B values across PWA were small, indicating relatively weak connections induced by the task. Despite this, clear moderate to moderately strong relationships were discovered between spared cortical tissue and the connection parameters across each family. In terms of input strength, trending associations showed that the more spared tissue in LIFG and LMTG, the greater the task-induced driving strength was for those regions for families #1 and #3, respectively (*r* = 0.550, *p* = 0.051 and *r* = 0.538, *p* = 0.058) (see **Figure 8**). In general, these results demonstrate that more preserved cortical tissue in the ROIs was associated with more negative task-induced coupling of certain connections between all regions but greater and more positive strength of driving input of “classic” language regions.

#### Relationship between Connectivity Parameters and Behavioral Accuracy

**FIGURE 9:**
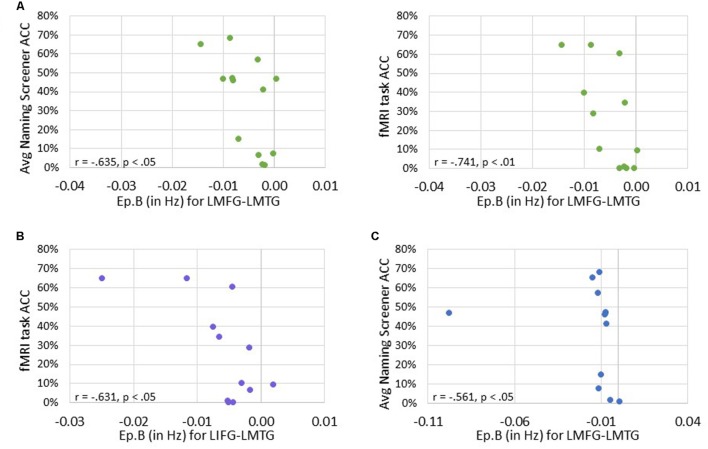
**Correlations between behavioral performance and strength of the connections (i.e., Ep.B values in Hz). (A)** For family #1, significant correlations were found between the connection strength of LMFG → LMTG and behavioral accuracy on the naming screener (shown on the left) and the fMRI task (shown on the right). **(B)** For family #2, a significant correlation was found between the connection strength of LIFG → LMTG and fMRI task accuracy. **(C)** For family #3, a significant correlation was found between the connection strength of LMFG → LMTG and the average naming screener accuracy.

Next, we investigated the relationship between connectivity parameters and behavioral performance on the fMRI task and the averaged picture naming screener accuracy. For family #1 (i.e., input to LIFG), significant moderately strong negative correlations were found between naming task accuracy and the connection from LMFG to LMTG. Specifically, the greater the accuracy on the baseline naming screeners and the fMRI task, the more negative the task-induced coupling was from LMFG to LMTG (*r* = -0.635, *p* < 0.05 and *r* = -0.741, *p* < 0.01, respectively). For family #2 (i.e., input to LMFG), higher fMRI task accuracy was significantly associated with more negative coupling from LIFG to LMTG (r = -0.631, *p* < 0.05). For family #3 (i.e., input to LMTG), higher accuracy on the baseline naming screeners was associated with more negative coupling from LMFG to LMTG (*r* = -0.561, *p* < 0.05). Once again, when outliers that may have influenced the results (see **Figure 9**) were removed from the analyses, the association between fMRI task accuracy and the modulatory connection from LIFG to LMTG for family #2 was no longer significant (*r* = -0.523, *p* = 0.099) but the moderate association between screener accuracy and the connection from LMFG to LMTG for family #3 remained (*r* = -0.580, *p* < 0.048). In the discussion, we will return to the rationale for including or excluding outliers from the analyses.

Significant positive associations were found between driving input strength and naming task accuracy such that greater task-induced perturbation of LIFG (in family #1) was significantly associated with higher accuracy on both the baseline picture naming screeners (*r* = 0.688, *p* < 0.01) and the fMRI task (*p* = 0.765, *p* < 0.01). Similarly, greater driving strength of LMFG (in family #2) was associated with greater fMRI task accuracy (*r* = 0.610, *p* < 0.05).

**FIGURE 10:**
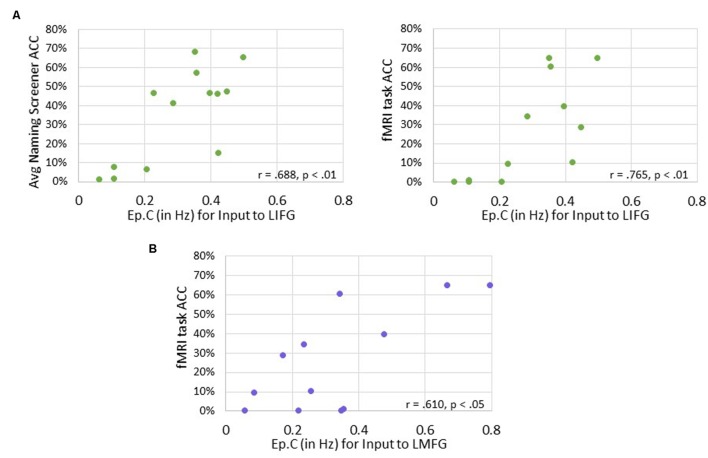
**Correlations between behavioral accuracy and strength of task-induced perturbation to specific regions (i.e., Ep.C values in Hz). (A)** For family #1, significant associations were found between strength of task-induced perturbation to LIFG and accuracy on the naming screener (shown on the left) and on the fMRI task (shown on the right). **(B)** For family #2, a significant relationship was seen between strength of driving input to LMFG and fMRI task accuracy.

Overall, greater task accuracy was related to more negative coupling between connections (especially the connection from LMFG to LMTG) (see **Figure 9**) while greater accuracy was related to greater and more positive driving input strength (see **Figure 10**). These findings suggest that network parameters are specifically associated with naming abilities for the items contained in the picture naming screener and the fMRI task. Also, the general trends of these results are similar to those seen in the correlations between connectivity parameters and spared cortical tissue.

## Discussion

The aims of the current study were twofold. First, we investigated how effective connectivity of a frontotemporal network induced by a picture naming task differed between neurologically intact participants and PWA. Second, as we were most interested in cortical reorganization in the PWA group, we examined the relationships between connectivity parameters, the amount of spared tissue in each region of interest, and behavioral performance. In order to investigate the effects of task-induced exogenous input to and all possible connections between LIFG, LMFG, and LMTG, the DCMs for the current study were constructed and then partitioned into three families organized by driving input to these regions. The family of models with driving input to LMTG (i.e., family #3) were based on the neurocognitive literature which indicates that picture naming is a semantically driven task and semantic processing is mediated by LMTG (e.g., Indefrey and Levelt, 2000, 2004; Indefrey, 2011). Therefore, the models from this family were constructed from a framework of possible connectivity observed during normal processing, and we hypothesized that this family of models would best fit the control participant data. Conversely, families with driving input to LIFG and LMFG (i.e., families #1 and #2, respectively) were modeled after the literature indicating that prefrontal regions are essential for selection and/or executive control processes and that these regions can mediate activation in other neural areas in a top-down fashion for challenging cognitive tasks. Specifically, LIFG is believed to mediate domain-specific processes of semantic and/or phonological selection or control while LMFG may be considered to mediate more domain-general control processes. Therefore, we hypothesized that activation would be driven by models with input to either LIFG (i.e., family #1) or LMFG (i.e., family #2) for PWA because they struggled with the picture naming task due to their impairments.

Contrary to expectations, the group-level BMS results revealed that the best-fit family of models for control participants was family #1 (i.e., input to LIFG). While somewhat surprising, it is possible these results align with the literature citing that greater demands on top–down control processes are seen for healthy older adults due to natural deterioration of neural structures with age (Meinzer et al., 2009, 2012; Park and Reuter-Lorenz, 2009). Alternatively, it may be the nature of the fMRI task, not the age of the control participants, which resulted in heavier reliance on LIFG for this group. Both behavioral (e.g., Howard et al., 2006) and neuroimaging (e.g., Janssen et al., 2011) studies have found that naming items within the same semantic category can result in semantic interference, which can manifest as decreased accuracy and/or increased response latency. Furthermore, Schnur et al. (2009) discovered that resolution of conflict between semantically related words was associated solely with activation in LIFG. As the fMRI task in the current study required participants to name items from within only three semantic categories, it is possible that driving input to LIFG was paramount to successful lexical selection by resolving competition between many active lexical representations for this group (e.g., Thompson-Schill et al., 1997).

For the group level PWA data, the best-fit family of models was family #2 (i.e., input to LMFG), which was in line with our initial hypothesis. One way to interpret this finding is based on the functional role LMFG plays during processing. As referenced previously, researchers agree that LMFG, in conjunction with other brain regions, comprises a neural network essential for domain-general cognitive flexibility and control. First and foremost, one may question whether LMFG should even be considered for inclusion within the language network. Fedorenko and Thompson-Schill (2014) address this very issue, stating that in the literature, the term “language network” frequently includes “classic” regions within the lateral left frontal and temporal cortices as well as regions contained within the bilateral domain-general cognitive-control network. The extent to which language processing is functionally specialized is contested in the normal processing literature and is even further complicated in the context of brain damage. For PWA, it is possible that domain-general regions subsume some of the responsibilities of damaged “classic” language regions. In the case of the current study, PWA activation for the picture naming task is best modeled by domain-general regions influencing activation in spared tissue in domain-specific regions.

Another way to interpret the group-level BMS results is that models with driving input to LMFG best fit the PWA data because LMFG was relatively spared across the group. At the single-subject level, this hypothesis holds true for certain participants (i.e., PWA 2, PWA 17) but not for all. For example, PWA 3 has a lesion that is nearly entirely confined to posterior regions, and consequently, damage to LMTG is great while both LIFG and LMFG are relatively spared. However, models with driving input to LMFG fit this individual’s data best even though theoretically, LIFG was available to assume its given role. Even more striking are the BMS results for PWA 9. This individual’s lesion is small with minimal damage to any of the three ROIs, yet the family of models with driving input to LMFG also fit this participant’s data the best. While the model-level inferences regarding LMFG are interesting, these results are not informative regarding *how* LMFG (or LIFG/LMTG) functions for PWA within the network.

Therefore, in line with the second goal of the study, we delved more deeply into the potential interaction between dynamic connectivity and spared tissue and behavior. The main findings from our analyses are summarized in **Table 5.**

**Table 5:** Summary of correlations between connectivity parameters and spared cortical tissue and behavior.

	Ep.C			Ep.B					
		
	LIFG	LMFG	LMTG	LIFG-LMFG	LIFG-LMTG	LMFG-LIFG	LMFG-LMTG	LMTG-LIFG	LMTG-LMFG
% Spared tissue LIFG	(+)					(-)			
% Spared tissue LMFG						(-)	(-)		
% Spared tissue LMTG			(+)					(+)	(-)
fMRI task ACC	(+)	(+)			(-)		(-)		
Naming screener ACC	(+)						(-)(-)		

*Colored cells indicate significant results; striped cells indicate associations that approached significance. The color of a cell reflects the family from which the connectivity parameter involved in the correlation was gleaned: green, family #1 (i.e., input to LIFG); purple, family #2 (i.e., input to LMFG); and blue, family #3 (input to LMTG). (-) indicates a negative relationship while (+) indicates the relationship was positive.*

Turning first to the results involving strength of driving regions (i.e., Ep.C), we found trending associations between the amount of spared cortical tissue in LIFG and LMTG and the strength of the task-induced perturbation to these regions. These results align well with our hypotheses, as one might assume that activation in core language regions would be impacted by a picture naming task if a greater portion of tissue in those regions is preserved. Somewhat less expected, however, is that greater spared tissue in LIFG and LMFG (not LMTG) was significantly associated with greater accuracy on the fMRI task. This finding again validates the importance of LMFG within the PWA neural network for picture naming as LMFG acts as a driving force in activation for individuals who perform better on the task. Furthermore, the collective associations with Ep.C values also highlight the importance of LIFG within the patient language network. Essentially, greater preservation of LIFG is associated with greater picture naming task-induced activation in that region as well as better naming performance. Therefore, it can be inferred that greater recruitment of LIFG relates to better language abilities, which is a finding regarding this region that has been cited not only the activation literature (e.g., Turkeltaub et al., 2011) but also in a recent connectivity study regarding treatment-induced reorganization of language networks in PWA by our group (Kiran et al., 2015).

A more complex picture emerges when considering the results involving coupling parameters between regions (Ep.B). First, it should be noted that our final conclusions pertain to results that include the individual (i.e., PWA 9) who appears as an outlier in **Figures 7** and **9.** Of note, this PWA has a relatively small lesion and mild aphasia yet his anomia is still pronounced, as indicated by his accuracy on the fMRI task, his picture naming screener accuracy, and his BNT score. Despite the heterogeneity in aphasia severity, lesion size and location, and naming abilities with the PWA group, the common characteristic of all PWA in the study was the presence of anomia. Therefore, this individual is representative of the group and his inclusion in these analyses is justified. Second, broadly speaking, greater spared cortical tissue in a given region was significantly related to greater modulatory effects for a connection that included that region. For example, greater spared tissue in LIFG and LMFG was related to the strength of a connection between those two regions (i.e., LMFG-LIFG). While cortical damage and functional connectivity are not mutually exclusive (Cloutman and Lambon Ralph, 2012), a greater degree of connectivity between highly intact neural hubs makes sense. Another general trend in these results is regarding the direction of the relationships between Ep.B values and spared tissue and behavior. The one exception to this trend was a positive association seen such that more spared tissue in LMTG was related to a nearly null effect of task on the connection from LMTG to LIFG for family #2. Interestingly, there was significantly less coupling of this connection for PWA relative to controls, so it possible that latent effects of damage to LIFG and/or disconnection between these cortical regions contributed to the minimal coupling from LMTG to LIFG induced by the task.

Most of the aforementioned associations were negative. In other words, more preserved tissue and better performance on the naming tasks was associated with more inhibitory connections between regions. Initially, these results may seem counterintuitive but a pattern with respect to LMFG emerges that may provide some insight into the role this region has for the task. Essentially, the more PWA resembled controls in terms of behavior and cortical integrity, the more LMFG inhibited activation in either LIFG or LMTG, yet that is not to say these results reflected a pattern that would be observed in controls. First, it is important to keep in mind that PWA with relatively high behavioral performance and minimal damage to these three regions do not have intact neural networks, and these individuals still struggled with the picture naming task. Furthermore, the inter-regional inhibitory influences were relatively weak. It is therefore possible that rather than inhibiting all neural activity, LMFG had more of a regulatory role on activation in the other regions in order to maximize success for the task.

Overall, this study highlights the importance of the role of LMFG within the language network for PWA and contributes to a limited literature linking measures of cortical integration, integrity, and behavior in this population. To our knowledge, very few studies have investigated these relationships in PWA using effective connectivity methods. In one such study, Papoutsi et al. (2011) found that better syntactic performance in a group of PWA was associated with enhanced connectivity between inferior frontal and mid-posterior temporal regions as well as increased integrity of the arcuate fasciculus and the extreme capsule system. A handful of other studies have investigated the relationships between either functional or structural connectivity of frontotemporal regions, structural integrity of the cortices and/or underlying white matter pathways, and behavior in PWA. For example, Han et al. (2013) discovered that lesion volume and fractional anisotropy of the left IFOF, anterior thalamic, and left UF tracts correlated with performance across three different types of semantic tasks, including oral picture naming. In line with the present study, these authors also analyzed the relative influence of gray matter lesions on performance and found that after controlling for a variety of additional factors (e.g., percentage of lesioned white matter tract tissue, general cognitive abilities), the only two significant associations were between semantic composite scores and preserved cortical tissue in LIFG and LMTG. Bonilha et al. (2014) examined the relationship between cortical necrosis, cortical disconnection, and behavioral measures of naming performance and global language skills. They found that models that included both damage to specific regions and disconnection of those regions to other cortical regions (e.g., BA45/pars opercularis of LIFG) were significantly related to language abilities (e.g., confrontation naming abilities) whereas models that included just necrotic damage to cortical regions were not related to language skills. Therefore, while it would be prudent in the future to also incorporate measures of white matter tract integrity, the results of the present study are in agreement with the findings of these previous investigations with regards to the importance of certain cortical regions in language processing (i.e., LIFG, LMTG) as well as the relationship between structural integrity and performance.

While the present study provides some new insights into brain-behavior relationships in chronic aphasia, it is important to consider some additional factors when interpreting the results. First, DCM is a hypothesis-driven method of effective connectivity analysis that precludes exploratory or data-driven means of investigating connectivity. Therefore, the particular neural regions were selected *a priori* based on the PWA activation literature, and the model space was constructed to specifically investigate differences in frontotemporal connections during picture naming according to driving input to regions. It is certain that different results would have been obtained if additional regions had been included in the model space and/or if the model space had been specified differently.

Furthermore, creation of the VOIs for the DCM analysis was based on gross rather than fine-grained parcellation of neural regions although different portions of these regions have been implicated in different neurophysiological functions. For example, some researchers (e.g., Poldrack et al., 1999; Bokde et al., 2001; Binder et al., 2009; Binder and Desai, 2011) have suggested that ventral regions of LIFG (i.e., BA 47/pars orbitalis and BA 45/pars triangularis) support semantic processing while more dorsal regions of LIFG (i.e., BA 45/pars triangularis and BA 44/pars opercularis) support phonological processing. On the other hand, other researchers (e.g., Thompson-Schill et al., 1997; Wagner et al., 2001; Gold and Buckner, 2002; Vigneau et al., 2006) have found activation associated with controlled use of semantic and phonological information in all three parts of LIFG. Similarly, mid-LMTG is believed to mediate multi-modal semantic processing while posterior LMTG has been implicated in word form retrieval and semantic control, and the bilateral ATLs are thought to be an amodal semantic store (Indefrey and Levelt, 2004; Patterson et al., 2007; Binney et al., 2010; Visser et al., 2010, 2012; Whitney et al., 2011; Jefferies, 2013). However, the overarching rationale for not selecting VOIs based on finer parceled regions or more stringent anatomical criteria is twofold. First, in order to complete group-level analyses in DCM, each region must be modeled individually for each participant. Variability in lesion location precluded group-level spatial localization of peak maxima in the PWA group, and limiting selection of the VOIs to specific subregions would have restricted the PWA pool. To stay consistent across participant groups, the same selection criteria were applied to the control data. Second, relatively little is certainly known regarding how perilesional regions functionally reorganize in PWA. Fridriksson (2010) suggests that rebuilt language networks in PWA do not necessarily resemble language networks in neurologically intact individuals; rather, neighboring neural regions that have the potential to subserve a specific language function may subsume that function in light of brain damage (Zahn et al., 2006). For some PWA, a single activated cluster of voxels was seen in certain regions, especially those individuals with minimal spared tissue in a given region. Therefore, it is possible that such a node was essential for a given language function but shifted due to brain damage. Consequently, it would be imprudent to exclude these data and remove those individuals from the analysis.

Moving forward, there are several open avenues of research that remain untraversed regarding how task-based effective connectivity, structural damage and behavioral performance are related. Expanding the number of cortical regions to other left hemisphere language regions and the right hemisphere homologues would provide further insight into the entire language network and how right hemisphere connectivity may be either compensatory or maladaptive for PWA. Furthermore, given the inherent heterogeneity within aphasia in general and within the present sample in particular, it may be practical to investigate effective connectivity at a single-subject level (e.g., construct DCMs including regions crucial for the task of interest for each participant) and then make connections between different aphasia profiles according to lesion and behavioral characteristics. Lastly, many recent studies (e.g., Saur et al., 2008; Turken and Dronkers, 2011; Bonilha et al., 2014; Duffau et al., 2014) have highlighted the contribution of damage to white matter tracts to language deficits. Therefore, examining the association between task-based effective connectivity and both white and gray matter integrity may be the best way to elucidate a more complete picture of brain function-structure relationships. Thus far, however, the results from the present study demonstrate that frontotemporal effective connectivity for picture naming differs between neurologically intact healthy controls and individuals with chronic aphasia and that connectivity in PWA is related both to performance and to the extent of cortical damage.

## Author Contributions

EM took the lead on conceptualizing the project and designing the effective connectivity analyses. EM assisted with acquisition and analysis of the behavioral and fMRI data and conducted all of the effective connectivity analyses. EM took the lead on interpreting the results and writing the manuscript. KK led fMRI data acquisition and assisted with analysis of the fMRI data for the majority of the participants. KK also contributed to the design and execution of the effective connectivity analyses. KK assisted in drafting the manuscript, especially the imaging and connectivity methods, and provided comments and revised drafts of the manuscript. SK designed the fMRI task and served as a scientific mentor to EM throughout the study. SK contributed significantly to the conceptualization of the project, selection of appropriate methods, and interpretation of the results. SK also contributed significantly to the writing of the manuscript.

## Conflict of Interest Statement

The authors declare that the research was conducted in the absence of any commercial or financial relationships that could be construed as a potential conflict of interest.

## Footnotes

1 http://cnlr.northwestern.edu/.

2 http://websites.psychology.uwa.edu.au/school/MRCDatabase/uwa_mrc.htm

3 https://www.nitrc.org/projects/mricron

4 http://www.alivelearn.net/xjview
